# Some Properties of Fuzzy Soft Proximity Spaces

**DOI:** 10.1155/2015/752634

**Published:** 2015-01-27

**Authors:** İzzettin Demir, Oya Bedre Özbakır

**Affiliations:** ^1^Department of Mathematics, Duzce University, 81620 Duzce, Turkey; ^2^Department of Mathematics, Ege University, 35100 Izmir, Turkey

## Abstract

We study the fuzzy soft proximity spaces in Katsaras's sense. First, we show how a fuzzy soft topology is derived from a fuzzy soft proximity. Also, we define the notion of fuzzy soft *δ*-neighborhood in the fuzzy soft proximity space which offers an alternative approach to the study of fuzzy soft proximity spaces. Later, we obtain the initial fuzzy soft proximity determined by a family of fuzzy soft proximities. Finally, we investigate relationship between fuzzy soft proximities and proximities.

## 1. Introduction

In 1999, Molodtsov [1] initiated the concept of soft set theory as a new approach for coping with uncertainties and also presented the basic results of the new theory. This new theory does not require the specification of a parameter. We can utilize any parametrization with the aid of words, sentences, real numbers, and so on. This implies that the problem of setting the membership function does not arise. Hence, soft set theory has compelling applications in several diverse fields; most of these applications were shown by Molodtsov [1]. Nowadays, there are a lot of works related to soft set theory and its applications [2–9].

Fuzzy soft set which is a combination of fuzzy set and soft set was introduced by Maji et al. [10]. Roy and Maji [11] gave some results on an application of fuzzy soft sets in decision making problem. Then, Tanay and Kandemir [12] initiated the notion of fuzzy soft topology and gave some fundamental properties of it by following Chang [13]. Also, the fuzzy soft topology in Lowen's sense [14] was given by Varol and Aygün [15]. Fuzzy soft sets and their applications have been investigated intensively in recent years [16–24].

Proximity structure was introduced by Efremovic in 1951 [25, 26]. It can be considered either as axiomatizations of geometric notions or as suitable tools for an investigation of topology. Moreover, this structure has a very significant role in many problems of topological spaces such as compactification and extension problems. The most comprehensive work on the theory of proximity spaces was done by Naimpally and Warrack [27]. Then, many authors have obtained the concept of fuzzy proximity structure in different approaches. By using fuzzy sets, Katsaras [28] defined fuzzy proximity and studied the relation with fuzzy topology in Chang's sense. Later, Artico and Moresco [29, 30] proposed a new definition of fuzzy proximity on a set *X* as a map *δ* : *L* ^*X*^ × *L* ^*X*^ → {0,1} satisfying certain conditions, where *L* is a completely distributive lattice with an order reversing involution. In connection with a fuzzy topology in [31], the different notion of a fuzzy proximity was introduced by Markin and Sostak [32]. In 2005, Ramadan et al. [33] presented fuzzifying proximity structures.

Extensions of proximity structures to the soft sets and also fuzzy soft sets have been studied by some authors. More recently, Hazra et al. [34] defined soft proximity spaces and studied some of their properties. By using fuzzy soft sets, Çetkin et al. [35] introduced soft fuzzy proximity spaces on the base of the axioms suggested by Markin and Sostak [32] and Katsaras [28], respectively. All these works have generalized versions of many of the well-known results on proximity spaces.

Motivated by their works, we continue investigating the properties of fuzzy soft proximity spaces in Katsaras's sense. We show that each fuzzy soft proximity *δ* on *X* induces a fuzzy soft topology *τ*(*δ*) on the same set. Also, we define the notion of fuzzy soft *δ*-neighborhood in a fuzzy soft proximity space and obtain a few results analogous to the ones that hold for *δ*-neighborhood in proximity spaces. We prove the existences of initial fuzzy soft proximity structures. Based on this fact, we introduce products of fuzzy soft proximity spaces. The relation between a fuzzy soft proximity and a proximity is also investigated.

## 2. Preliminaries

In this section, we recall some basic notions regarding fuzzy soft sets which will be used in the sequel. Throughout this work, let *X* be an initial universe, *I* ^*X*^ be the set of all fuzzy subsets of *X* and *E* be the set of all parameters for *X*.

**Definition 1 (see [10]).**

A fuzzy soft set *f* on the universe *X* with the set *E* of parameters is defined by the set of ordered pairs

(1) $$\begin{array}{c} f=\left\{\left(e,f\left(e\right)\right):e\in E,f\left(e\right)\in I^{X}\right\}, \end{array}$$

where *f* is a mapping given by *f* : *E* → *I* ^*X*^.

Throughout this paper, the family of all fuzzy soft sets over *X* is denoted by *FS*(*X*, *E*).

**Definition 2 (see [10, 15, 16]).**

Let *f*, *g* ∈ *FS*(*X*, *E*). Then, we have the following.

- The fuzzy soft set *f* is called null fuzzy soft set, denoted by $\tilde{\emptyset }$, if *f*(*e*) = 0_*X*_ for every *e* ∈ *E*.
- If *f*(*e*) = 1_*X*_, for all *e* ∈ *E*, then *f* is called absolute fuzzy soft set, denoted by $\tilde{X}$.
- *f* is a fuzzy soft subset of *g* if *f*(*e*) ≤ *g*(*e*) for each *e* ∈ *E*. It is denoted by *f*⊑*g*.
- *f* and *g* are equal if *f*⊑*g* and *g*⊑*f*. It is denoted by *f* = *g*.
- The complement of *f* is denoted by *f* ^*c*^, where *f* ^*c*^ : *E* → *I* ^*X*^ is a mapping defined by *f* ^*c*^(*e*) = 1_*X*_ − *f*(*e*) for all *e* ∈ *E*. Clearly, (*f* ^*c*^)^*c*^ = *f*.
- The union of *f* and *g* is a fuzzy soft set *h* defined by *h*(*e*) = *f*(*e*)∨*g*(*e*) for all *e* ∈ *E*. *h* is denoted by *f*⊔*g*.
- The intersection of *f* and *g* is a fuzzy soft set *h* defined by *h*(*e*) = *f*(*e*)∧*g*(*e*) for all *e* ∈ *E*. *h* is denoted by *f*⊓*g*.

**Definition 3 (see [16]).**

Let *J* be an arbitrary index set and let {*f*_*i*_}_*i*∈*J*_ be a family of fuzzy soft sets over *X*. Then,

- the union of these fuzzy soft sets is the fuzzy soft set *h* defined by *h*(*e*) = ∨_*i*∈*J*_ *f*_*i*_(*e*) for every *e* ∈ *E* and this fuzzy soft set is denoted by ⊔_*i*∈*J*_ *f*_*i*_;
- the intersection of these fuzzy soft sets is the fuzzy soft set *h* defined by *h*(*e*) = ∧_*i*∈*J*_ *f*_*i*_(*e*) for every *e* ∈ *E* and this fuzzy soft set is denoted by ⊓_*i*∈*J*_ *f*_*i*_.

**Theorem 4 (see [15]).**

Let *J* be an index set and *f*, *g*, *f*_*i*_, *g*_*i*_ ∈ *FS*(*X*, *E*), for all *i* ∈ *J*. Then, the following statements are satisfied:

1. *f*⊓(⊔_*i*∈*J*_⁡*g*_*i*_) = ⊔_*i*∈*J*_(*f*⊓*g*_*i*_);
2. *f*⊔(⊓_*i*∈*J*_ *g*_*i*_) = ⊓_*i*∈*J*_(*f*⊔*g*_*i*_);
3. (⊓_*i*∈*J*_ *f*_*i*_)^*c*^ = ⊔_*i*∈*J*_ *f*_*i*_^*c*^;
4. (⊔_*i*∈*J*_ *f*_*i*_)^*c*^ = ⊓_*i*∈*J*_ *f*_*i*_^*c*^;
5. if *f*⊑*g*, then *g* ^*c*^⊑*f* ^*c*^.

**Definition 5 (see [18]).**

A fuzzy soft set *f* over *X* is said to be a fuzzy soft point if there is an *e* ∈ *E* such that *f*(*e*) is a fuzzy point in *X* (i.e., there exists an *x* ∈ *X* such that *f*(*e*)(*x*) = *α* ∈ (0,1] and *f*(*e*)(*x*′) = 0 for all *x*′ ∈ *X* − {*x*}) and *f*(*e*′) = 0_*X*_ for every *e*′ ∈ *E*∖{*e*}. It will be denoted by *e*_*x*^*α*^_.

The fuzzy soft point *e*_*x*^*α*^_ is said to belong to a fuzzy soft set *f*, denoted by $e_{x^{\alpha }}\tilde{\in }f$, if *α* ≤ *f*(*e*)(*x*).

**Definition 6 (see [20]).**

Let *FS*(*X*, *E*) and *FS*(*Y*, *K*) be the families of all fuzzy soft sets over *X* and *Y*, respectively. Let *φ* : *X* → *Y* and *ψ* : *E* → *K* be two mappings. Then, the mapping *φ*_*ψ*_ is called a fuzzy soft mapping from *X* to *Y*, denoted by *φ*_*ψ*_ : *FS*(*X*, *E*) → *FS*(*Y*, *K*).

(1) Let *f* ∈ *FS*(*X*, *E*). Then *φ*_*ψ*_(*f*) is the fuzzy soft set over *Y* defined as follows:
  (2) φψ(f)(k)(y)={⋁x∈φ−1(y)(⋁e∈ψ−1(k)f(e))(x),if  ψ−1(k)≠∅, φ−1(y)≠∅;0,otherwise,
 for all *k* ∈ *K* and all *y* ∈ *Y*.
 
*φ*_*ψ*_(*f*) is called an image of a fuzzy soft set *f*.
(2) Let *g* ∈ *FS*(*Y*, *K*). Then *φ*_*ψ*_^−1^(*g*) is the soft set over *X* defined as follows:
  (3) $$\begin{array}{c} \varphi_{\psi }^{-1}\left(g\right)\left(e\right)\left(x\right)=g\left(\psi \left(e\right)\right)\left(\varphi \left(x\right)\right) \end{array}$$
 for all *e* ∈ *E* and all *x* ∈ *X*.
 
*φ*_*ψ*_^−1^(*g*) is called a preimage of a fuzzy soft set *g*.

The fuzzy soft mapping *φ*_*ψ*_ is called injective, if *φ* and *ψ* are injective. The fuzzy soft mapping *φ*_*ψ*_ is called surjective, if *φ* and *ψ* are surjective.

**Theorem 7 (see [20]).**

Let *f*_*i*_ ∈ *FS*(*X*, *E*) and *g*_*i*_ ∈ *FS*(*Y*, *K*) for all *i* ∈ *J*, where *J* is an index set. Then, for a fuzzy soft mapping *φ*_*ψ*_ : *FS*(*X*, *E*) → *FS*(*Y*, *K*), the following conditions are satisfied:

1. if *f*_1_⊑*f*_2_, then *φ*_*ψ*_(*f*_1_)⊑*φ*_*ψ*_(*f*_2_);
2. if *g*_1_⊑*g*_2_, then *φ*_*ψ*_^−1^(*g*_1_)⊑*φ*_*ψ*_^−1^(*g*_2_);
3. *φ*_*ψ*_(⊔_*i*∈*J*_ *f*_*i*_) = ⊔_*i*∈*J*_ *φ*_*ψ*_(*f*_*i*_);
4. *φ*_*ψ*_(⊓_*i*∈*J*_ *f*_*i*_)⊑⊓_*i*∈*J*_ *φ*_*ψ*_(*f*_*i*_);
5. *φ*_*ψ*_^−1^(⊔_*i*∈*J*_ *g*_*i*_) = ⊔_*i*∈*J*_ *φ*_*ψ*_^−1^(*g*_*i*_);
6. *φ*_*ψ*_^−1^(⊓_*i*∈*J*_ *g*_*i*_) = ⊓_*i*∈*J*_ *φ*_*ψ*_^−1^(*g*_*i*_);
7. $\varphi_{\psi }^{-1}(\tilde{Y})=\tilde{X},\varphi_{\psi }^{-1}(\tilde{\emptyset })=\tilde{\emptyset }$;
8. $\varphi_{\psi }(\tilde{\emptyset })=\tilde{\emptyset }$.

**Theorem 8 (see [15, 18]).**

Let *f*, *f*_*i*_ ∈ *FS*(*X*, *E*) for all *i* ∈ *J*, where *J* is an index set, and let *g* ∈ *FS*(*Y*, *K*). Then, for a fuzzy soft mapping *φ*_*ψ*_ : *FS*(*X*, *E*) → *FS*(*Y*, *K*), the following conditions are satisfied:

1. *f*⊑*φ*_*ψ*_^−1^(*φ*_*ψ*_(*f*)), and the equality holds if *φ*_*ψ*_ is injective;
2. *φ*_*ψ*_(*φ*_*ψ*_^−1^(*g*))⊑*g*, and the equality holds if *φ*_*ψ*_ is surjective;
3. *φ*_*ψ*_(⊓_*i*∈*J*_ *f*_*i*_) = ⊓_*i*∈*J*_ *φ*_*ψ*_(*f*_*i*_) if *φ*_*ψ*_ is injective;
4. $\varphi_{\psi }(\tilde{X})=\tilde{Y}$ if *φ*_*ψ*_ is surjective.

**Definition 9 (see [15]).**

Let *f* ∈ *FS*(*X*, *E*) and *g* ∈ *FS*(*Y*, *K*). The fuzzy soft product *f* × *g* is defined by the fuzzy soft set *h* where *h* : *E* × *K* → *I* ^*X*×*Y*^ and *h*(*e*, *k*) = *f*(*e*) × *g*(*k*) for all (*e*, *k*) ∈ *E* × *K*.

**Definition 10 (see [15]).**

Let *f* ∈ *FS*(*X*, *E*) and *g* ∈ *FS*(*Y*, *K*) and let *p*_*X*_ : *X* × *Y* → *X*, *q*_*E*_ : *E* × *K* → *E* and *p*_*Y*_ : *X* × *Y* → *Y*, *q*_*K*_ : *E* × *K* → *K* be the projection mappings in classical meaning. The fuzzy soft mappings (*p*_*X*_)_*q*_*E*__ and (*p*_*Y*_)_*q*_*K*__ are called fuzzy soft projection mappings from *X* × *Y* to *X* and from *X* × *Y* to *Y*, respectively, where (*p*_*X*_)_*q*_*E*__(*f* × *g*) = *f* and (*p*_*Y*_)_*q*_*K*__(*f* × *g*) = *g*.

**Theorem 11 .**

Every parameterized collection of fuzzy subsets in *X* is a fuzzy soft set. Also, every fuzzy soft set is a parameterized collection of fuzzy subsets in some universe.

**Proof**

Consider any parameterized collection {*μ*_*α*_ : *α* ∈ Δ} of fuzzy subsets in *X*. Then, *f* : Δ → *I* ^*X*^ defined by *f*(*α*) = *μ*_*α*_ is a fuzzy soft set over *X*.

**Definition 12 (see [12]).**

Let *τ* be the collection of fuzzy soft sets over *X*; then *τ* is said to be a fuzzy soft topology on *X* if

(*fst*_1_) $\tilde{\emptyset },\tilde{X}$ belong to *τ*,
(*fst*_2_) the union of any number of fuzzy soft sets in *τ* belongs to *τ*,
(*fst*_3_) the intersection of any two fuzzy soft sets in *τ* belongs to *τ*.

(*X*, *τ*) is called a fuzzy soft topological space. The members of *τ* are called fuzzy soft open sets in *X*. A fuzzy soft set *f* over *X* is called a fuzzy soft closed in *X* if *f* ^*c*^ ∈ *τ*.

**Example 13 .**

Let *X* = *I* and *E* = (0,1). Let us consider the following fuzzy soft sets on *X* with the set *E* of parameters:

(4) f(e)(x)={0,if  0≤x≤e;x−e,if  e≤x≤1WWWiWW∀e∈E, all  x∈X,g(e)(x)={e−x,if  0≤x≤e;0,if  e≤x≤1WWWWiW∀e∈E, all  x∈X.

Then, $\tau =\{\tilde{\emptyset },\tilde{X},f,g,f⊔g\}$ is a fuzzy soft topology on *X*.

**Definition 14 (see [12]).**

Let (*X*, *τ*) be a fuzzy soft topological space and *f* ∈ *FS*(*X*, *E*). The fuzzy soft interior of *f* is the fuzzy soft set *f* ^*o*^ = ⊔{*g* : *g*  is  a  fuzzy  soft  open  set  and  *g*⊑*f*}.

By property (*fst*_2_) for fuzzy soft open sets, *f* ^*o*^ is fuzzy soft open. It is the largest fuzzy soft open set contained in *f*.

**Definition 15 (see [15, 17]).**

Let (*X*, *τ*) be a fuzzy soft topological space and *f* ∈ *FS*(*X*, *E*). The fuzzy soft closure of *f* is the fuzzy soft set $\bar{f}=⊓\{g:g\;\text{is}\;\text{a}\;\text{fuzzy}\;\text{soft}\;\text{closed}\;\text{set}\;\text{and}\;f⊑g\}$.

Clearly $\bar{f}$ is the smallest fuzzy soft closed set over *X* which contains *f*.

**Theorem 16 (see [15]).**

Let us consider an operator associating with each fuzzy soft set *f* on *X* another fuzzy soft set $\bar{f}$ such that the following properties hold:

(*fo*_1_) $f⊑\bar{f}$,
(*fo*_2_) $\bar{\bar{f}}=\bar{f}$,
(*fo*_3_) $\bar{f\vee g}=\bar{f}\vee \bar{g}$,
(*fo*_4_) $\bar{\tilde{\emptyset }}=\tilde{\emptyset }$.

Then, the family

(5) $$\begin{array}{c} \tau =\left\{f\in FS\left(X,E\right):\bar{f^{c}}=f^{c}\right\} \end{array}$$

defines a fuzzy soft topology on *X* and, for every *f* ∈ *FS*(*X*, *E*), the fuzzy soft set $\bar{f}$ is the fuzzy soft closure of *f* in the fuzzy soft topological space (*X*, *τ*).

This operator is called the fuzzy soft closure operator.

**Definition 17 (see [15, 18]).**

Let (*X*, *τ*_1_) and (*Y*, *τ*_2_) be two fuzzy soft topological spaces. A fuzzy soft mapping *φ*_*ψ*_ : (*X*, *τ*_1_)→(*Y*, *τ*_2_) is called fuzzy soft continuous if *φ*_*ψ*_^−1^(*g*) ∈ *τ*_1_ for every *g* ∈ *τ*_2_.

**Theorem 18 (see [15]).**

Let (*X*, *τ*) be a fuzzy soft topological space, where *τ* = {*f*_*α*_ : *α* ∈ Δ}. Then, the collection *τ*_*e*_ = {*f*_*α*_(*e*)∣*α* ∈ Δ} for every *e* ∈ *E* defines a fuzzy topology on *X*.

**Theorem 19 .**

Every parameterized collection of fuzzy topological spaces on *X* determines a fuzzy soft topological space over *X*.

**Proof**

Let {(*X*, *τ*_*e*_) : *e* ∈ *E*} be a parameterized family of fuzzy topological spaces. Let us define a fuzzy soft topological space (*X*, *τ*) as the following: let *τ* be the collection of all the mappings *f*, where *f* : *E* → *I* ^*X*^ such that *f*(*e*) ∈ *τ*_*e*_ for each *e* ∈ *E*. Then, *τ* is a fuzzy soft topology on *X*. Indeed,

(*fst*_1_) $\tilde{\emptyset }\in \tau$, because $\tilde{\emptyset }:E\to I^{X}$ and $\tilde{\emptyset }(e)=0_{X}\in \tau_{e}$ for each *e* ∈ *E*; similarly, $\tilde{X}\in \tau$, because $\tilde{X}:E\to I^{X}$ and $\tilde{X}(e)=1_{X}\in \tau_{e}$ for each *e* ∈ *E*;
(*fst*_2_) let {*f*_*i*_∣*i* ∈ *J*} be a collection of members in *τ*; then, for all *i* ∈ *J*, we have *f*_*i*_(*e*) ∈ *τ*_*e*_ for each *e* ∈ *E*; therefore, ⊔_*i*∈*J*_ *f*_*i*_ is a mapping ⊔_*i*∈*J*_ *f*_*i*_ : *E* → *I* ^*X*^ such that (⊔_*i*∈*J*_ *f*_*i*_)(*e*) = ∨_*i*∈*J*_ *f*_*i*_(*e*) ∈ *τ*_*e*_ for each *e* ∈ *E*; consequently, ⊔_*i*∈*J*_ *f*_*i*_ ∈ *τ*;
(*fst*_3_) let *f*, *g* ∈ *τ*; then, *f*(*e*), *g*(*e*) ∈ *τ*_*e*_ for each *e* ∈ *E* and hence *f*⊓*g* is a mapping *f*⊓*g* : *E* → *I* ^*X*^ such that (*f*⊓*g*)(*e*) = *f*(*e*)∧*g*(*e*) ∈ *τ*_*e*_ for each *e* ∈ *E*; thus, *f*⊓*g* ∈ *τ*.

Recall that a binary relation *δ* on the power set of a set *X* is called a proximity on *X* if the following axioms are satisfied (see, [27]):

(*p*_1_) $\emptyset \bar{\delta }A$;
(*p*_2_) if *A*∩*B* ≠ *∅*, then *AδB*;
(*p*_3_) if *AδB*, then *BδA*;
(*p*_4_) *Aδ*(*B* ∪ *C*) if and only if *AδB* or *AδC*;
(*p*_5_) if $A\bar{\delta }B$, then there exists a subset *C* of *X* such that $A\bar{\delta }C$ and $B\bar{\delta }(X-C)$,

where $\bar{\delta }$ means negation of *δ*.

The pair (*X*, *δ*) is called a proximity space; two subsets *A* and *B* of the set *X* are close with respect to *δ* if *AδB*; otherwise, they are remote with respect to *δ*.

**Definition 20 (see [28]).**

A binary relation *δ* on *I* ^*X*^ is called a fuzzy proximity if *δ* satisfies the following conditions:

(*fp*_1_) $0_{X}\bar{\delta }\mu$;
(*fp*_2_) if *μ*∧*ρ* ≠ 0_*X*_, then *μδρ*;
(*fp*_3_) if *μδρ*, then *ρδμ*;
(*fp*_4_) *μδ*(*ρ*∨*σ*) if and only if *μδρ* or *μδσ*;
(*fp*_5_) if $\mu \bar{\delta }\rho$, then there exists a *σ* ∈ *I* ^*X*^ such that $\mu \bar{\delta }\sigma$ and $\rho \bar{\delta }(1_{X}-\sigma )$.

A fuzzy proximity space is a pair (*X*, *δ*) comprising a set *X* and a fuzzy proximity *δ* on the set *X*.

## 3. Fuzzy Soft Proximities

In this section, we study some elementary properties of fuzzy soft proximity structures in Katsaras's sense. We induce a fuzzy soft topology from a given fuzzy soft proximity by using the fuzzy soft closure operator. Also, we present an alternative description of the concept of fuzzy soft proximity, which is called fuzzy soft *δ*-neighborhood.

**Definition 21 (see [35]).**

A mapping *δ* : *K* → 2^*FS*(*X*,*E*)×*FS*(*X*,*E*)^ is called a Katsaras (*E*, *K*)-soft fuzzy proximity on a set *X*, where *E* and *K* are arbitrary nonempty sets viewed on the sets of parameters, if, for any *f*, *g*, *h* ∈ *FS*(*X*, *E*) and *k* ∈ *K*, the following conditions are satisfied:

(*sfp*_1_) $\tilde{\emptyset }\bar{\delta_{k}}f$;
(*sfp*_2_) if $f\bar{\delta_{k}}g$, then $f⊑(\tilde{X}-g)$;
(*sfp*_3_) if *fδ*_*k*_ *g*, then *gδ*_*k*_ *f*;
(*sfp*_4_) *fδ*_*k*_(*g*⊔*h*) if and only if *fδ*_*k*_ *g* or *fδ*_*k*_ *h*;
(*sfp*_5_) if $f\bar{\delta_{k}}g$, then there exists an *h* ∈ *FS*(*X*, *E*) such that $f\bar{\delta_{k}}h$ and $g\bar{\delta_{k}}(\tilde{X}-h)$.

The pair (*X*, *δ*) is called a Katsaras (*E*, *K*)-soft fuzzy proximity space, where, for every *k* ∈ *K*, *δ*_*k*_ ⊂ *FS*(*X*, *E*) × *FS*(*X*, *E*) is a relation on *FS*(*X*, *E*).

The following definition coincides with Definition 21 when the parameter set *K* is a singleton set.

**Definition 22 .**

A binary relation *δ* ⊂ *FS*(*X*, *E*) × *FS*(*X*, *E*) is called a fuzzy soft proximity on *X* if *δ* satisfies the following conditions:

(*fsp*_1_) $\tilde{\emptyset }\bar{\delta }f$;
(*fsp*_2_) if $f⊓g\neq \tilde{\emptyset }$, then *fδg*;
(*fsp*_3_) if *fδg*, then *gδf*;
(*fsp*_4_) *fδ*(*g*⊔*h*) if and only if *fδg* or *fδh*;
(*fsp*_5_) if $f\bar{\delta }g$, then there exists an *h* ∈ *FS*(*X*, *E*) such that $f\bar{\delta }h$ and $g\bar{\delta }(\tilde{X}-h)$.

The pair (*X*, *δ*) is called a fuzzy soft proximity space.

**Example 23 .**

On any set *X*, let us define *fδg* if and only if $f\neq \tilde{\emptyset }$ and $g\neq \tilde{\emptyset }$. This defines a fuzzy soft proximity on *X*.

We have easily the following Lemma.

**Lemma 24 .**

If (*X*, *δ*) is a fuzzy soft proximity space, then it satisfies the following properties:

- if *fδg* and *k*⊒*f*, *h*⊒*g*, then *kδh*;
- *fδf* for each $f\neq \tilde{\emptyset }$;
- $f\delta \tilde{X}$ if and only if $f\neq \tilde{\emptyset }$.

Let (*X*, *δ*) be a fuzzy soft proximity space. For every *f* ∈ *FS*(*X*, *E*), we define

(6) $$\begin{array}{c} \bar{f}=\tilde{X}-⊔\left\{g\in FS\left(X,E\right):f\bar{\delta }g\right\}. \end{array}$$

Then we get the following theorem.

**Theorem 25 .**

Let (*X*, *δ*) be a fuzzy soft proximity space. Then, the mapping $f\to \bar{f}$ satisfies the conditions (*fo*_1_)–(*fo*_4_). Therefore, the collection

(7) $$\begin{array}{c} \tau \left(\delta \right)=\left\{f\in FS\left(X,E\right):\bar{f^{c}}=f^{c}\right\} \end{array}$$

is a fuzzy soft topology on *X*.

**Proof**

We will show that the mapping $f\to \bar{f}$ has the properties (*fo*_1_)–(*fo*_4_).

(*fo*_1_) Suppose that $f\neq \tilde{\emptyset }$. Let *e* ∈ *E* and *x* ∈ *X*. Take any *g* ∈ *FS*(*X*, *E*) such that $g\bar{\delta }f$. Then, by (*fsp*_2_), $g⊓f=\tilde{\emptyset }$. Hence, either *g*(*e*)(*x*) = 0 or *f*(*e*)(*x*) = 0. In both cases, we obtain *g*(*e*)(*x*) ≤ 1 − *f*(*e*)(*x*). Therefore, $\vee_{g\bar{\delta }f}g(e)(x)\leq 1-f(e)(x)$. Thus, we get

(8) $$\begin{array}{c} f\left(e\right)\left(x\right)\leq 1-\underset{g\bar{\delta }f}{⋁}g\left(e\right)\left(x\right)=\bar{f}\left(e\right)\left(x\right). \end{array}$$

(*fo*_2_) It is enough to show that *gδf* if and only if $g\delta \bar{f}$. Necessity follows immediately from Lemma 24. For sufficiency, let $g\delta \bar{f}$. Suppose that $g\bar{\delta }f$. Then, by (*fsp*_5_), there is an *h* ∈ *FS*(*X*, *E*) such that $g\bar{\delta }h$ and $f\bar{\delta }(\tilde{X}-h)$. Because $g\bar{\delta }h$ and $g\delta \bar{f}$, there exist an *e* ∈ *E* and an *x* ∈ *X* such that $h(e)(x)<\bar{f}(e)(x)$. Now, we will choose number *a*, where $h(e)(x)<a<\bar{f}(e)(x)$ and let us define *e*_*x*^1−*a*^_ ∈ *FS*(*X*, *E*). Since 1 − *a* ≤ 1 − *h*(*e*)(*x*), we have $e_{x^{1-a}}⊑\tilde{X}-h$. Also, *e*_*x*^1−*a*^_ *δf*, since, otherwise, we would have $\bar{f}(e)(x)\leq 1-(1-a)=a$ which is impossible. By *e*_*x*^1−*a*^_ *δf* and $e_{x^{1-a}}⊑(\tilde{X}-h)$, $(\tilde{X}-h)\delta f$. This is a contradiction to the fact that $f\bar{\delta }(\tilde{X}-h)$.

(*fo*_3_) It is easy to verify that $\bar{f⊔g}⊒\bar{f}⊔\bar{g}$. Conversely, suppose that there exist an *e* ∈ *E* and an *x* ∈ *X* such that $\bar{f⊔g}(e)(x)>\bar{f}(e)(x)\vee \bar{g}(e)(x)$. Take an *ϵ* > 0 satisfying

(9) $$\begin{array}{c} a=\bar{f⊔g}\left(e\right)\left(x\right)>\bar{f}\left(e\right)\left(x\right)\vee \bar{g}\left(e\right)\left(x\right)+\epsilon . \end{array}$$

We may assume $\bar{f}(e)(x)\geq \bar{g}(e)(x)$ (the case $\bar{g}(e)(x)>\bar{f}(e)(x)$ is analogous). Then, since $\bar{f}(e)(x)=1-\vee \{h(e)(x):h\bar{\delta }f\}<a-\epsilon$, there exists an *h* ∈ *FS*(*X*, *E*) such that $h\bar{\delta }f$ and 1 − *h*(*e*)(*x*) < *a* − *ϵ*. By the inequality $1-h(e)(x)\geq \bar{f}(e)(x)\geq \bar{g}(e)(x)>\bar{g}(e)(x)-\epsilon /2$, we have $1-h(e)(x)+\epsilon /2>\bar{g}(e)(x)$. Because $\bar{g}(e)(x)=1-\vee \{k(e)(x):k\bar{\delta }g\}$, there exists a *k* ∈ *FS*(*X*, *E*) such that $k\bar{\delta }g$ and *h*(*e*)(*x*) − *ϵ*/2 < *k*(*e*)(*x*). Now, since $(h⊓k)\bar{\delta }f$ and $(h⊓k)\bar{\delta }g$, we obtain $\left(h⊓k)\bar{\delta }(\bar{f⊔g}\right)$. From (*fsp*_2_), it follows that $\bar{f⊔g}(e)(x)\leq 1-(h⊓k)(e)(x)$. Also, we get *h*(*e*)(*x*) − *ϵ*/2 < (*k*⊓*h*)(*e*)(*x*). Thus,

(10) a=f⊔g¯(e)(x)≤1−(h⊓k)(e)(x)≤1−h(e)(x)+ϵ2<a−ϵ+ϵ2=a−ϵ2,

which yields a contradiction.

(*fo*_4_) Because of $\tilde{\emptyset }\bar{\delta }\tilde{X}$, we get $\bar{\tilde{\emptyset }}=\tilde{\emptyset }$

Trivially, the fuzzy soft proximity space defined in Example 23 induces the fuzzy soft topological space $\tau =\{\tilde{\emptyset },\tilde{X}\}$.

**Definition 26 .**

Let (*X*, *δ*) be a fuzzy soft proximity space. For *f*, *g* ∈ *I* ^*X*^, the fuzzy soft set *g* is said to be a fuzzy soft *δ*-neighborhood of *f* if $f\bar{\delta }(\tilde{X}-g)$; we write this in symbols as *f*⋐*g*.

**Theorem 27 .**

Let (*X*, *δ*) be a fuzzy soft proximity space. Then the relation ⋐ satisfies the following properties:

(*fspn*_1_) $\tilde{\emptyset }⋐f$;
(*fspn*_2_) *f*⋐*g* implies $\left(\tilde{X}-g)⋐(\tilde{X}-f\right)$;
(*fspn*_3_) *f*⋐*g* implies $f⊓g^{c}=\tilde{\emptyset }$;
(*fspn*_4_) *f*⋐(*g*⊓*h*) if and only if *f*⋐*g* and *f*⋐*h*;
(*fspn*_5_) *f*⊑*g*⋐*h*⊑*k* implies *f*⋐*k*;
(*fspn*_6_) *f*⋐*g* implies there is an *h* ∈ *FS*(*X*, *E*) such that *f*⋐*h*⋐*g*.

**Proof**

(*fspn*_1_) is obvious.

(*fspn*_2_) If *f*⋐*g*, then $f\bar{\delta }(\tilde{X}-g)$. By (*fsp*_3_), $(\tilde{X}-g)\bar{\delta }f$; that is, $\tilde{X}-g⋐\tilde{X}-f$.

(*fspn*_3_) Let *f*⋐*g*. Then, from (*fsp*_2_), it follows that $f⊓g^{c}=\tilde{\emptyset }$.

(*fspn*_4_) Consider *f*⋐*g*⊓*h* $\Leftrightarrow f\bar{\delta }{\left(g⊓h\right)}^{c}=g^{c}⊔h^{c}$ $\Leftrightarrow f\bar{\delta }g^{c}$ and $f\bar{\delta }h^{c}$⇔*f*⋐*g* and *f*⋐*h*.

(*fspn*_5_) If $f\bar{⋐}k$, where $\bar{⋐}$ means negation of ⋐, then $f\delta (\tilde{X}-k)$. Since *f*⊑*g* and $\tilde{X}-k⊑\tilde{X}-h$, we have $g\delta (\tilde{X}-h)$. Therefore, $g\bar{⋐}h$, which is a contradiction.

(*fspn*_6_) Consider that *f*⋐*g* implies $f\bar{\delta }(\tilde{X}-g)$. Then, by (*fsp*_5_), there exists an *h* ∈ *FS*(*X*, *E*) such that $f\bar{\delta }(\tilde{X}-h)$ and $h\bar{\delta }(\tilde{X}-g)$. Hence, *f*⋐*h*⋐*g*.

**Theorem 28 .**

Let (*X*, *δ*) be a fuzzy soft proximity space and *f*, *g* ∈ *FS*(*X*, *E*). Then, the following statements are satisfied:

- *f*⋐*g* if and only if $\bar{f}⋐g$;
- if *f*⋐*g*, then there is a *k* ∈ *τ*(*δ*) such that *f*⊑*k*⊑*g*;
- if $f\bar{\delta }g$, then there exist fuzzy soft sets *h*, *k* such that *f*⋐*h*, *g*⋐*k*, and $h\bar{\delta }k$.

**Proof**

(i) It is clear by the fact that *gδf* if and only if $g\delta \bar{f}$ (see the proof of Theorem 25).

(ii) Let *f*⋐*g*. Then, $f\bar{\delta }(\tilde{X}-g)$ and this implies that

(11) $$\begin{array}{c} \bar{\tilde{X}-g}=\tilde{X}-⊔\left\{h:\left(\tilde{X}-g\right)\bar{\delta }h\right\}⊑\tilde{X}-f. \end{array}$$

Set $k=\tilde{X}-(\bar{\tilde{X}-g})$. It is easy to verify that

(12) $$\begin{array}{c} \bar{\tilde{X}-k}=\bar{\bar{\tilde{X}-g}}=\bar{\tilde{X}-g}=\tilde{X}-k. \end{array}$$

Thus, we obtain *k* ∈ *τ*(*δ*) and *f*⊑*k*⊑*g*.

(iii) If $f\bar{\delta }g$, then, from (*fsp*_5_), there is a fuzzy soft set *k* such that $f\bar{\delta }k$ and $g\bar{\delta }(\tilde{X}-k)$. Because $k\bar{\delta }f$, there is a fuzzy soft set *h* such that $k\bar{\delta }h$ and $f\bar{\delta }(\tilde{X}-h)$. Thus, there exist fuzzy soft sets *h* and *k* such that *f*⋐*h*, *g*⋐*k*, and $h\bar{\delta }k$.

**Theorem 29 .**

Let ⋐ be a relation on *FS*(*X*, *E*) satisfying (*fspn*_1_)−(*fspn*_6_). Then, *δ* is a fuzzy soft proximity on *X* defined as follows:

(13) $$\begin{array}{c} f\bar{\delta }g\;iff\;f⋐\left(\tilde{X}-g\right). \end{array}$$

Also, according to this fuzzy soft proximity, *g* is a fuzzy soft *δ*-neighbourhood of *f* if and only if *f*⋐*g*.

**Proof**

We first need to verify axioms (*fsp*_1_)–(*fsp*_5_).

(*fsp*_1_) Let *f* ∈ *FS*(*X*, *E*). By (*fspn*_1_), we have $\tilde{\emptyset }⋐(\tilde{X}-f)$ and thus $\tilde{\emptyset }\bar{\delta }f$.
(*fsp*_2_) Let $f\bar{\delta }g$. Then, *f*⋐*g* ^*c*^ and from (*fspn*_3_) it follows that
  (14) $$\begin{array}{c} f⊓g=f⊓{\left(g^{c}\right)}^{c}=\tilde{\emptyset }. \end{array}$$
(*fsp*_3_) If $f\bar{\delta }g$, then *f*⋐*g* ^*c*^. By (*fspn*_2_), *g*⋐*f* ^*c*^ and hence $g\bar{\delta }f$.
(*fsp*_4_) Consider $f\bar{\delta }(g⊔h)\Leftrightarrow f⋐(\tilde{X}-(g⊔h))$⇔$f⋐(\tilde{X}-g)$ and $f⋐(\tilde{X}-h)$⇔$f\bar{\delta }g$ and $f\bar{\delta }h$.
(*fsp*_5_) Let $f\bar{\delta }g$. Then $f⋐(\tilde{X}-g)$. Therefore, by (*fspn*_6_), there is a fuzzy soft set *h* such that $f⋐h⋐(\tilde{X}-g)$. Thus, $f\bar{\delta }(\tilde{X}-h)$ and $h\bar{\delta }g$.

Hence, *δ* is a fuzzy soft proximity on *X*. From the definitions of the terms involved, it follows easily that *g* is a fuzzy soft *δ*-neighbourhood of *f* if and only if *f*⋐*g*.

**Theorem 30 .**

If (*X*, *δ*) is a fuzzy soft proximity space and *f* ∈ *FS*(*X*, *E*), then

(15) $$\begin{array}{c} \bar{f}=⊓\left\{g:f⋐g\right\}. \end{array}$$

**Proof**

Let us take a fuzzy soft set *g* such that *f*⋐*g*. Therefore, $\bar{f}⋐g$ and by (*fspn*_3_) we obtain $\bar{f}⊑g$. Hence,

(16) $$\begin{array}{c} \bar{f}⊑⊓\left\{g:f⋐g\right\}. \end{array}$$

On the other hand, suppose that there are an *e* ∈ *E* and an *x* ∈ *X* such that $\wedge \{g(e)(x):f⋐g\}>\bar{f}(e)(x)$. Let ∧{*g*(*e*)(*x*) : *f*⋐*g*} = *a*. Then there exists an *ϵ* > 0 such that

(17) $$\begin{array}{c} \bar{f}\left(e\right)\left(x\right)=1-\vee \left\{h\left(e\right)\left(x\right):f\bar{\delta }h\right\}<a-\epsilon . \end{array}$$

Therefore, there exists a fuzzy soft set *k* such that $f\bar{\delta }k$ and 1 − *k*(*e*)(*x*) < *a* − *ϵ*. Because $f\bar{\delta }k$, we have $f⋐(\tilde{X}-k)$. Hence, $⊓\{g:f⋐g\}⊑(\tilde{X}-k)$. Thus,

(18) $$\begin{array}{c} a=\wedge \left\{g\left(e\right)\left(x\right):f⋐g\right\}\leq 1-k\left(e\right)\left(x\right)<a-\epsilon , \end{array}$$

which leads to a contradiction.

**Definition 31 (see [35]).**

Let (*X*, *δ*_1_) and (*Y*, *δ*_2_) be two fuzzy soft proximity spaces. A fuzzy soft mapping *φ*_*ψ*_ : (*X*, *δ*_1_)→(*Y*, *δ*_2_) is a fuzzy soft proximity mapping if it satisfies

(19) $$\begin{array}{c} f\delta_{1}g⟹\varphi_{\psi }\left(f\right)\delta_{2}\varphi_{\psi }\left(g\right) \end{array}$$

for every *f*, *g* ∈ *FS*(*X*, *E*).

Using the above definition, we can easily prove the following propositions.

**Proposition 32 .**

Let (*X*, *δ*_1_) and (*Y*, *δ*_2_) be two fuzzy soft proximity spaces. A fuzzy soft mapping *φ*_*ψ*_ : (*X*, *δ*_1_)→(*Y*, *δ*_2_) is a fuzzy soft proximity mapping if and only if

(20) $$\begin{array}{c} h\bar{\delta_{2}}k⟹\varphi_{\psi }^{-1}\left(h\right)\bar{\delta_{1}}\varphi_{\psi }^{-1}\left(k\right), \end{array}$$

or in another form

(21) $$\begin{array}{c} h{⋐}_{2}k⟹\varphi_{\psi }^{-1}\left(h\right){⋐}_{1}\varphi_{\psi }^{-1}\left(k\right), \end{array}$$

for every *h*, *k* ∈ *FS*(*Y*, *K*).

**Proposition 33 .**

The composition of two fuzzy soft proximity mappings is a fuzzy soft proximity mapping.

**Theorem 34 .**

A fuzzy soft proximity mapping *φ*_*ψ*_ : (*X*, *δ*_1_)→(*Y*, *δ*_2_) is fuzzy soft continuous with respect to *τ*(*δ*_1_) and *τ*(*δ*_2_).

**Proof**

Let *f* ∈ *τ*(*δ*_2_). Now let us take any *h* ∈ *FS*(*Y*, *K*) such that $h\bar{\delta_{2}}(\tilde{Y}-f)$. Since *φ*_*ψ*_ is a fuzzy soft proximity mapping, we obtain $\varphi_{\psi }^{-1}(h)\bar{\delta_{1}}(\tilde{X}-\varphi_{\psi }^{-1}(f))$. From (*fsp*_2_), it follows that $\bar{\tilde{X}-\varphi_{\psi }^{-1}(f)}⊑\tilde{X}-\varphi_{\psi }^{-1}(h)$. Then, for every *e* ∈ *E* and every *x* ∈ *X*, we have

(22) (X~−φψ−1(f))¯(e)(x)≤(X~−φψ−1(h))(e)(x)=1−h(ψ(e))(φ(x))=(Y~−h)(ψ(e))(φ(x)).

Therefore,

(23) (X~−φψ−1(f))¯(e)(x)WW≤(⨅hδ2¯(Y~−f)⁡(Y~−h))(ψ(e))(φ(x))WW=(Y~−f¯)(ψ(e))(φ(x))WW=(Y~−f)(ψ(e))(φ(x))WW=1−φψ−1(f)(e)(x)WW=(X~−φψ−1(f))(e)(x).

Thus, since $\bar{\tilde{X}-\varphi_{\psi }^{-1}(f)}⊑\tilde{X}-\varphi_{\psi }^{-1}(f)$, we obtain *φ*_*ψ*_^−1^(*f*) ∈ *τ*(*δ*_1_).

**Definition 35 .**

If *δ*_1_ and *δ*_2_ are two fuzzy soft proximities on *X*, we define

(24) $$\begin{array}{c} \delta_{1}<\delta_{2}\;\text{iff}\;f\delta_{2}g\;\text{implies}\;f\delta_{1}g. \end{array}$$

The above is expressed by saying that *δ*_2_ is finer than *δ*_1_, or *δ*_1_ is coarser than *δ*_2_.

## 4. Initial Fuzzy Soft Proximities

We prove the existences of initial fuzzy soft proximity structure. Based on this fact, we define the product of fuzzy soft proximity spaces.

**Definition 36 .**

Let *X* be a set and {(*X*_*α*_, *δ*_*α*_) : *α* ∈ Δ} a family of fuzzy soft proximity spaces, and, for each *α* ∈ Δ, let (*φ*_*ψ*_)_*α*_ : *FS*(*X*, *E*)→(*X*_*α*_, *δ*_*α*_) be a fuzzy soft mapping. The initial structure *δ* is the coarsest fuzzy soft proximity on *X* for which all mappings (*φ*_*ψ*_)_*α*_ : (*X*, *δ*)→(*X*_*α*_, *δ*_*α*_)  (*α* ∈ Δ) are fuzzy soft proximity mapping.

**Theorem 37 (existence of initial structures).**

Let *X* be a set {(*X*_*α*_, *δ*_*α*_) : *α* ∈ Δ} a family of fuzzy soft proximity spaces, and, for each *α* ∈ Δ, let (*φ*_*ψ*_)_*α*_ : *FS*(*X*, *E*)→(*X*_*α*_, *δ*_*α*_) be a fuzzy soft mapping. For any *f*, *g* ∈ *FS*(*X*, *E*), define *fδg* if and only if, for every finite families {*f*_*i*_ : *i* = 1,…, *n*} and {*g*_*j*_ : *j* = 1,…, *m*}, where *f* = ⊔_*i*=1_^*n*^ *f*_*i*_ and *g* = ⊔_*j*=1_^*m*^ *g*_*j*_, there exist an *f*_*i*_ and a *g*_*j*_ such that

(25) $$\begin{array}{c} {\left(\varphi_{\psi }\right)}_{\alpha }\left(f_{i}\right)\delta_{\alpha }{\left(\varphi_{\psi }\right)}_{\alpha }\left(g_{j}\right)\;foreach\;\alpha \in \Delta . \end{array}$$

Then *δ* is the coarsest fuzzy soft proximity on *X* for which all mappings (*φ*_*ψ*_)_*α*_ : (*X*, *δ*)→(*X*_*α*_, *δ*_*α*_)  (*α* ∈ Δ) are fuzzy soft proximity mapping.

**Proof**

We first prove that *δ* is a fuzzy soft proximity on *X*.

(*fsp*_1_) is obvious.
(*fsp*_2_) We will show that if $f\bar{\delta }g$, then $f⊓g=\tilde{\emptyset }$. Let $f\bar{\delta }g$. Then, there exist finite covers *f* = ⊔_*i*=1_^*n*^ *f*_*i*_ and *g* = ⊔_*j*=1_^*m*^ *g*_*j*_ of *f* and *g*, respectively, such that ${\left(\varphi_{\psi }\right)}_{\alpha }(f_{i})\bar{\delta_{\alpha }}{\left(\varphi_{\psi }\right)}_{\alpha }(g_{j})$ for some *α* = *s*_*ij*_ ∈ Δ, where *i* = 1,…, *n* and *j* = 1,…, *m*. Since each *δ*_*α*_ is a fuzzy soft proximity, ${\left(\varphi_{\psi }\right)}_{\alpha }(f_{i})⊓{\left(\varphi_{\psi }\right)}_{\alpha }(g_{j})=\tilde{\emptyset }$. From this, it follows that
  (26) (φψ)α(⨆i=1nfi)⊓(φψ)α(⨆j=1mgj)WW=(φψ)α(f)⊓(φψ)α(g)=∅~.
 Thus, we have $f⊓g=\tilde{\emptyset }$.
(*fsp*_3_) Since each *δ*_*α*_ is a fuzzy soft proximity, it is clear that *fδg* implies *gδf*.
(*fsp*_4_) It is easy to verify that if *fδg*, then *fδh* for each *h*⊒*g*. Therefore, *fδg* or *fδh* implies *fδ*(*g*⊔*h*). Conversely, assume that $f\bar{\delta }g$ and $f\bar{\delta }h$. Then, there exist finite covers *f* = ⊔_*i*=1_^*n*^ *f*_*i*_ and *g* = ⊔_*j*=1_^*m*^ *g*_*j*_ of *f* and *g*, respectively, such that ${\left(\varphi_{\psi }\right)}_{\alpha }(f_{i})\bar{\delta_{\alpha }}{\left(\varphi_{\psi }\right)}_{\alpha }(g_{j})$ for some *α* = *s*_*ij*_ ∈ Δ, where *i* = 1,…, *n* and *j* = 1,…, *m*. In the same way, there are finite covers *f* = ⊔_*p*=1_^*q*^ *k*_*p*_ and *h* = ⊔_*j*=*m*+1_^*m*+*l*^ *g*_*j*_ of *f* and *h*, respectively, such that ${\left(\varphi_{\psi }\right)}_{\alpha }(k_{p})\bar{\delta_{\alpha }}{\left(\varphi_{\psi }\right)}_{\alpha }(g_{j})$ for some *α* = *t*_*pj*_ ∈ Δ, where *p* = 1,…, *q* and *j* = *m* + 1,…, *m* + *l*. Now, *f* = ⊔{*f*_*i*_⊓*k*_*p*_ : *i* = 1,…, *n*; *p* = 1,…, *q*} and *g*⊔*h* = ⊔{*g*_*j*_ : *j* = 1,…, *m* + *l*} are finite covers of *f* and *g*⊔*h*, respectively. Hence, from the fact that ${\left(\varphi_{\psi }\right)}_{\alpha }(f_{i}⊓k_{p})\bar{\delta_{\alpha }}{\left(\varphi_{\psi }\right)}_{\alpha }(g_{j})$ for *α* = *s*_*ij*_ or *α* = *t*_*pj*_, it follows that $f\bar{\delta }(g⊔h)$.
(*fsp*_5_) Let us define the set *Ω* of all pairs (*f*, *g*) such that $f\bar{\delta }g$ and we have either *fδh* or $g\delta (\tilde{X}-h)$ for each *h* ∈ *FS*(*X*, *E*). The validity of (*fsp*_5_) will follow from the fact that *Ω* is empty. Suppose, on the contrary, that (*f*, *g*) ∈ *Ω*. Then, (*φ*_*ψ*_)_*α*_(*f*)*δ*_*α*_(*φ*_*ψ*_)_*α*_(*g*) for each *α* ∈ Δ. Indeed, let *h* ∈ *FS*(*X*_*α*_, *E*_*α*_) and *k* = (*φ*_*ψ*_)_*α*_^−1^(*h*). If *fδk*, then (*φ*_*ψ*_)_*α*_(*f*)*δ*_*α*_(*φ*_*ψ*_)_*α*_(*k*). Because (*φ*_*ψ*_)_*α*_(*k*)⊑*h*, we have (*φ*_*ψ*_)_*α*_(*f*)*δ*_*α*_ *h*. Similarly, if $g\delta (\tilde{X}-k)$, then ${\left(\varphi_{\psi }\right)}_{\alpha }(g)\delta_{\alpha }({\tilde{X}}_{\alpha }-h)$. Hence, since *δ*_*α*_ is a fuzzy soft proximity on *X*_*α*_, we obtain (*φ*_*ψ*_)_*α*_(*f*)*δ*_*α*_(*φ*_*ψ*_)_*α*_(*g*). Also, we observe that for each (*f*, *g*) ∈ *Ω* there are positive integers *n*, *m* and covers *f* = ⊔_*i*=1_^*n*^ *f*_*i*_ and *g* = ⊔_*j*=1_^*m*^ *g*_*j*_ such that, for every pair (*i*, *j*)∈{1,…, *n*}×{1,…, *m*}, there exists an *α* ∈ Δ satisfying ${\left(\varphi_{\psi }\right)}_{\alpha }(f_{i})\bar{\delta_{\alpha }}{\left(\varphi_{\psi }\right)}_{\alpha }(g_{j})$. Let *l* = *n* + *m*. It easy to see that *l* > 2. Then, for each (*f*, *g*) ∈ *Ω*, let us choose such an integer *l*. But *l* is not uniquely determined by (*f*, *g*). Let *κ* be the set of all integers corresponding to members of *Ω* and let *l* be the smallest member of *κ*. Take a (*f*, *g*) ∈ *Ω* such that *l* is the integer corresponding to it. Then, there are covers *f* = ⊔_*i*=1_^*n*^ *f*_*i*_ and *g* = ⊔_*j*=1_^*m*^ *g*_*j*_ such that *l* = *n* + *m* and for every pair (*i*, *j*)∈{1,…, *n*}×{1,…, *m*} and there exists an *α* ∈ Δ satisfying ${\left(\varphi_{\psi }\right)}_{\alpha }(f_{i})\bar{\delta_{\alpha }}{\left(\varphi_{\psi }\right)}_{\alpha }(g_{j})$. One of the *n*, *m* is greater than 1. Consider *n* > 1 and let *f*′ = *f*_1_⊔⋯⊔*f*_*n*−1_. In this case, one of the following conditions should be true:
  (i) for every *h* ∈ *FS*(*X*, *E*), either *f*′*δh* or $g\delta (\tilde{X}-h)$;
  (ii) for every *h* ∈ *FS*(*X*, *E*), either *f*_*n*_ *δh* or $g\delta (\tilde{X}-h)$.

In fact, suppose that neither (i) nor (ii) holds. Then, there are *h*_1_, *h*_2_ ∈ F*S*(*X*, *E*) such that $f^{\prime }\bar{\delta }h_{1},g\bar{\delta }(\tilde{X}-h_{1})$ and $f_{n}\bar{\delta }h_{2},g\bar{\delta }(\tilde{X}-h_{2})$. Letting *h* = *h*_1_⊓*h*_2_, we obtain $f\bar{\delta }h$ and $g\bar{\delta }(\tilde{X}-h)$, contradicting the fact that (*f*, *g*) ∈ *Ω*.

Suppose that (i) holds. Because *f*′⊑*f* and $f\bar{\delta }g$, this means that $f^{\prime }\bar{\delta }g$. Hence, by (i), we have (*f*′, *g*) ∈ *Ω*. But this is now a contradiction since (*n* − 1) + *m* = *l* − 1 ∈ *κ*, contrary to the choice of *l*. If (ii) holds, we get a contradiction in a similar way. Therefore, the set *Ω* is empty. Thus, *δ* is a fuzzy soft proximity on *X*.

It is easy to see that all mappings (*φ*_*ψ*_)_*α*_ : (*X*, *δ*)→(*X*_*α*_, *δ*_*α*_) are fuzzy soft proximity mapping. Let *δ* ^*^ be another fuzzy soft proximity on *X* making each of the mappings (*φ*_*ψ*_)_*α*_ : (*X*, *δ* ^*^)→(*X*_*α*_, *δ*_*α*_) fuzzy soft proximity mapping. We will show that *δ* < *δ* ^*^, which will complete the proof. Let *fδ* ^*^ *g* and consider any covers *f* = ⊔_*i*=1_^*n*^ *f*_*i*_ and *g* = ⊔_*j*=1_^*m*^ *g*_*j*_ of *f* and *g*, respectively. Since *f* = (*f*_1_⊔⋯⊔*f*_*n*_)*δ* ^*^ *g*, by (*fsp*_4_), there is an *i* ∈ {1,…, *n*} such that *f*_*i*_ *δg*. In the same way, since *f*_*i*_ *δ* ^*^ *g* = (*g*_1_⊔⋯⊔*g*_*m*_), by (*fsp*_4_), there is a *j* ∈ {1,…, *m*} such that *f*_*i*_ *δg*_*j*_. From the fact that all mappings (*φ*_*ψ*_)_*α*_ : (*X*, *δ* ^*^)→(*X*_*α*_, *δ*_*α*_) are fuzzy soft proximity mapping, it follows that (*φ*_*ψ*_)_*α*_(*f*_*i*_)*δ*_*α*_(*φ*_*ψ*_)_*α*_(*g*_*j*_) for each *α* ∈ Δ. Thus, we get *fδg*.

**Theorem 38 .**

A fuzzy soft mapping *φ*_*ψ*_ : (*Y*, *δ* ^*^)→(*X*, *δ*) is a fuzzy soft proximity mapping if and only if (*φ*_*ψ*_)_*α*_∘*φ*_*ψ*_ : (*Y*, *δ* ^*^)→(*X*_*α*_, *δ*_*α*_) is a fuzzy soft proximity mapping for every *α* ∈ Δ.

**Proof**

The necessity is easy. We prove the sufficiency. Suppose that (*φ*_*ψ*_)_*α*_∘*φ*_*ψ*_ is a fuzzy soft proximity mapping for every *α* ∈ Δ. Let *fδ* ^*^ *g* and let *φ*_*ψ*_(*f*) = ⊔_*i*=1_^*n*^ *f*_*i*_ and *φ*_*ψ*_(*g*) = ⊔_*j*=1_^*m*^ *g*_*j*_. Then, we have

(27) $$\begin{array}{c} f⊑{⨆}_{i=1}^{n}\varphi_{\psi }^{-1}\left(f_{i}\right),\;\;g⊑{⨆}_{j=1}^{m}{\left(\varphi_{\psi }\right)}^{-1}g_{j}. \end{array}$$

Since *fδ* ^*^ *g*, by (*fsp*_4_), there exist *i*, *j* such that *φ*_*ψ*_^−1^(*f*_*i*_)*δ* ^*^ *φ*_*ψ*_^−1^(*g*_*j*_). Because

(28) $$\begin{array}{c} {\left(\varphi_{\psi }\right)}_{\alpha }\circ \varphi_{\psi }\circ \varphi_{\psi }^{-1}\left(f_{i}\right)⊑\varphi_{\psi }\left(f_{i}\right),\\ {\left(\varphi_{\psi }\right)}_{\alpha }\circ \varphi_{\psi }\circ \varphi_{\psi }^{-1}\left(g_{j}\right)⊑\varphi_{\psi }\left(g_{j}\right), \end{array}$$

it follows from our hypothesis that (*φ*_*ψ*_)_*α*_(*f*_*i*_)*δ*_*α*_(*φ*_*ψ*_)_*α*_(*g*_*j*_) for every *α* ∈ Δ. This shows that *φ*_*ψ*_(*f*)*δφ*_*ψ*_(*g*).

**Definition 39 .**

Let {(*X*_*α*_, *δ*_*α*_) : *α* ∈ Δ} be a family of fuzzy soft proximity spaces and let *X* = ∏_*α*∈Δ_ *X*_*α*_ and *E* = ∏_*α*∈Δ_ *E*_*α*_ be product sets. An initial fuzzy soft proximity structure *δ* = ∏_*α*∈Δ_ *δ*_*α*_ on *X* with respect to all the fuzzy soft projection mappings (*p*_*X*_*α*__)_*q*_*E*_*α*___, where *p*_*X*_*α*__ : *X* → *X*_*α*_ and *q*_*E*_*α*__ : *E* → *E*_*α*_, is called the product fuzzy soft proximity structure.

(*X*, *δ*) is said to be a product fuzzy soft proximity space.

From Theorems 37 and 38, we obtain the following corollary.

**Corollary 40 .**

Consider {(*X*_*α*_, *δ*_*α*_) : *α* ∈ Δ} be a family of fuzzy soft proximity spaces. Let *X* = ∏_*α*∈Δ_ *X*_*α*_ and *E* = ∏_*α*∈Δ_ *E*_*α*_ be sets and for each *α* ∈ Δ let (*p*_*X*_*α*__)_*q*_*E*_*α*___ be a fuzzy soft mapping. For any *f*, *g* ∈ *FS*(*X*, *E*), define *fδg* if and only if, for every finite families {*f*_*i*_ : *i* = 1,…, *n*} and {*g*_*j*_ : *j* = 1,…, *m*}, where *f* = ⊔_*i*=1_^*n*^ *f*_*i*_ and *g* = ⊔_*j*=1_^*m*^ *g*_*j*_, there exist an *f*_*i*_ and a *g*_*j*_ such that (*p*_*X*_*α*__)_*q*_*E*_*α*___(*f*_*i*_)*δ*_*α*_(*p*_*X*_*α*__)_*q*_*E*_*α*___(*g*_*j*_) for each *α* ∈ Δ. Then,

- *δ* = ∏_*α*∈Δ_ *δ*_*α*_ is the coarsest fuzzy soft proximity on *X* for which all mappings (*p*_*X*_*α*__)_*q*_*E*_*α*___  (*α* ∈ Δ) are fuzzy soft proximity mapping;
- a fuzzy soft mapping *φ*_*ψ*_ : (*Y*, *δ* ^*^)→(*X*, *δ*) is a fuzzy soft proximity mapping if and only if (*p*_*X*_*α*__)_*q*_*E*_*α*___∘*φ*_*ψ*_ : (*Y*, *δ* ^*^)→(*X*_*α*_, *δ*_*α*_) is a fuzzy soft proximity mapping for every *α* ∈ Δ.

## 5. Fuzzy Soft Proximities Induced by Proximities

Our task in this section is to study the connection between fuzzy soft proximity spaces and proximity spaces.

**Definition 41 (see [17]).**

Let *X* be a set and let *A* be a subset of *X*. Then, a mapping ${\tilde{\chi }}_{A}:E\to I^{X}$ is a fuzzy soft set on *X* defined as the following:

(29) $$\begin{array}{c} {\tilde{\chi }}_{A}\left(e\right)=\chi_{A}\;\text{for}\;\text{every}\;e\in E, \end{array}$$

where *χ*_*A*_ is a characteristic function of *A*.

**Example 42 .**

Let *X* = {*x*_1_, *x*_2_, *x*_3_}, *A* = {*x*_1_, *x*_2_}, and *E* = {*e*_1_, *e*_2_}. Then,

(30) $$\begin{array}{c} {\tilde{\chi }}_{A}=\left\{\left(e_{1},\left\{\frac{x_{1}}{1},\frac{x_{2}}{1},\frac{x_{3}}{0}\right\}\right),\left(e_{2},\left\{\frac{x_{1}}{1},\frac{x_{2}}{1},\frac{x_{3}}{0}\right\}\right)\right\} \end{array}$$

is a fuzzy soft set on *X*.

**Lemma 43 .**

For any subsets *A*, *B* of *X*,

- ${\tilde{\chi }}_{A}⊓{\tilde{\chi }}_{B}={\tilde{\chi }}_{A\cap B}$;
- ${\tilde{\chi }}_{A}⊔{\tilde{\chi }}_{B}={\tilde{\chi }}_{A\cup B}$;
- ${\left({\tilde{\chi }}_{A}\right)}^{c}={\tilde{\chi }}_{A^{c}}$.

**Proof**

Straightforward.

**Theorem 44 .**

Let (*X*, *δ*) be a proximity space. By letting, for *f*, *g* ∈ *FS*(*X*, *E*),

$f\bar{\delta^{i}}g$ if and only if there exist subsets *A*, *B* of *X* such that $f⊑{\tilde{\chi }}_{A}$, $g⊑{\tilde{\chi }}_{B}$, and $A\bar{\delta }B$, one defines a fuzzy soft proximity on *X*.

**Proof**

We will show that *δ* ^*i*^ satisfies axioms (*fsp*_1_)–(*fsp*_5_).

(*fsp*_1_) From $\tilde{\emptyset }⊑{\tilde{\chi }}_{\emptyset }$, $f⊑{\tilde{\chi }}_{X}$, and $\emptyset \bar{\delta }X$, it follows that $\tilde{\emptyset }\bar{\delta^{i}}f$.
(*fsp*_2_) Let $f\bar{\delta^{i}}g$. Then, there are subsets *A* and *B* of *X* such that $f⊑{\tilde{\chi }}_{A}$, $g⊑{\tilde{\chi }}_{B}$, and $A\bar{\delta }B$. By $A\bar{\delta }B$, we have *A*∩*B* = *∅*, so that ${\tilde{\chi }}_{A}⊓{\tilde{\chi }}_{B}=\tilde{\emptyset }$. Thus, we get $f⊓g=\tilde{\emptyset }$.
(*fsp*_3_) It is clear because $A\bar{\delta }B$ implies $B\bar{\delta }A$.
(*fsp*_4_) It is easy to see that if $f\bar{\delta^{i}}(g⊔h)$, then $f\bar{\delta^{i}}g$ and $f\bar{\delta^{i}}h$. Conversely, suppose that $f\bar{\delta^{i}}g$ and $f\bar{\delta^{i}}h$. Then, there exist subsets *A* and *B* of *X* such that $f⊑{\tilde{\chi }}_{A}$, $g⊑{\tilde{\chi }}_{B}$, and $A\bar{\delta }B$. Likewise, there exist subsets *C* and *D* of *X* such that $f⊑{\tilde{\chi }}_{C}$, $h⊑{\tilde{\chi }}_{D}$, and $C\bar{\delta }D$. Since $f⊑{\tilde{\chi }}_{A}⊓{\tilde{\chi }}_{C}={\tilde{\chi }}_{A\cap C}$,  $g⊔h⊑{\tilde{\chi }}_{B}⊔{\tilde{\chi }}_{D}={\tilde{\chi }}_{B\cup D}$, and $\left(A\cap C)\bar{\delta }(B\cup D\right)$, we conclude that $f\bar{\delta^{i}}(g⊔h)$.
(*fsp*_5_) If $f\bar{\delta^{i}}g$, then there are subsets *A* and *B* of *X* such that $f⊑{\tilde{\chi }}_{A}$, $g⊑{\tilde{\chi }}_{B}$, and $A\bar{\delta }B$. Since $A\bar{\delta }B$, by (*p*_5_), there is a *C*⊆*X* such that $A\bar{\delta }C$ and $B\bar{\delta }(X-C)$. Therefore, for a fuzzy soft set ${\tilde{\chi }}_{C}$, we obtain $f\bar{\delta^{i}}{\tilde{\chi }}_{C}$ and $g\bar{\delta^{i}}(\tilde{X}-{\tilde{\chi }}_{C})$, which completes the proof.

**Theorem 45 .**

Let (*X*, *δ* ^*^) be a fuzzy soft proximity space.

- There is a proximity relation *δ* on *X* such that *δ* ^*^ = *δ* ^*i*^.
- If $f\bar{\delta^{*}}g$, then there exist subsets *A* and *B* of *X* such that $f⊑{\tilde{\chi }}_{A}$, $g⊑{\tilde{\chi }}_{B}$, and ${\tilde{\chi }}_{A}\bar{\delta^{*}}{\tilde{\chi }}_{B}$
- The relation *AδB* if and only if ${\tilde{\chi }}_{A}\delta^{*}{\tilde{\chi }}_{B}$ is a proximity on *X*.

Then, (*a*) and (*b*) are equivalent and they imply (*c*).

**Proof**

The implication (a)⇒(b) is obvious.

To prove that (b)⇒(c), since the other axioms are readily verified, it is enough to show that *δ* satisfies (*p*_5_). Let $A\bar{\delta }B$ for any subsets *A*, *B* of *X*. Because ${\tilde{\chi }}_{A}\bar{\delta^{*}}{\tilde{\chi }}_{B}$, there is an *f* ∈ *FS*(*X*, *E*) such that ${\tilde{\chi }}_{A}\bar{\delta^{*}}f$ and ${\tilde{\chi }}_{B}\bar{\delta^{*}}(\tilde{X}-f)$. By hypothesis, there exist subsets *C*, *D*, *G*, *H* of *X* such that ${\tilde{\chi }}_{A}⊑{\tilde{\chi }}_{C}$, $f⊑{\tilde{X}}_{D}$, ${\tilde{\chi }}_{B}⊑{\tilde{X}}_{G}$, $\tilde{X}-f⊑{\tilde{\chi }}_{H}$, ${\tilde{\chi }}_{C}\bar{\delta^{*}}{\tilde{\chi }}_{D}$, and ${\tilde{\chi }}_{G}\bar{\delta^{*}}{\tilde{\chi }}_{H}$. Because $f⊑{\tilde{X}}_{D}$ and $\tilde{X}-f⊑{\tilde{\chi }}_{H}$, we have ${\tilde{\chi }}_{\left(X-D\right)}⊑{\tilde{\chi }}_{H}$. Now, from ${\tilde{\chi }}_{C}\bar{\delta^{*}}{\tilde{\chi }}_{D}$ and ${\tilde{\chi }}_{A}⊑{\tilde{\chi }}_{C}$, it follows that ${\tilde{\chi }}_{A}\bar{\delta^{*}}{\tilde{\chi }}_{D}$ and so $A\bar{\delta }D$. Likewise, since ${\tilde{\chi }}_{G}\bar{\delta^{*}}{\tilde{\chi }}_{H}$, ${\tilde{\chi }}_{B}⊑{\tilde{\chi }}_{G}$, and ${\tilde{\chi }}_{\left(X-D\right)}⊑{\tilde{\chi }}_{H}$, we have ${\tilde{\chi }}_{B}\bar{\delta^{*}}{\tilde{\chi }}_{\left(X-D\right)}$ and this implies that $B\bar{\delta }(X-D)$. Thus, *δ* has the axiom (*p*_5_).

In order to prove (b)⇒(a), let us define the binary relation *δ* on the power set of *X* as follows:

(31) $$\begin{array}{c} A\delta B\;\text{iff}\;{\tilde{\chi }}_{A}\delta^{*}{\tilde{\chi }}_{B}. \end{array}$$

We have shown that *δ* is a proximity on *X*. To complete the proof, it will suffice to prove that *δ* ^*^ = *δ* ^*i*^, that is, that *fδ* ^*i*^ *g* if and only if *fδ* ^*^ *g*. Assume that $f\bar{\delta^{*}}g$. Then, by hypothesis, there exist subsets *A* and *B* of *X* such that $f⊑{\tilde{\chi }}_{A}$, $g⊑{\tilde{\chi }}_{B}$, and ${\tilde{\chi }}_{A}\bar{\delta^{*}}{\tilde{\chi }}_{B}$. Since ${\tilde{\chi }}_{A}\bar{\delta^{*}}{\tilde{\chi }}_{B}$, by definition, we have $A\bar{\delta }B$. Thus, $f\bar{\delta^{i}}g$. On the other hand, suppose that $f\bar{\delta^{i}}g$. Using the definition of *δ* ^*i*^, we obtain subsets *A*, *B* of *X* such that $f⊑{\tilde{\chi }}_{A}$, $g⊑{\tilde{\chi }}_{B}$, and $A\bar{\delta }B$. Therefore, we get ${\tilde{\chi }}_{A}\bar{\delta^{*}}{\tilde{\chi }}_{B}$. Thus, since $f⊑{\tilde{\chi }}_{A}$, $g⊑{\tilde{\chi }}_{B}$, and ${\tilde{\chi }}_{A}\bar{\delta^{*}}{\tilde{\chi }}_{B}$, we obtain $f\bar{\delta^{*}}g$ and the proof is concluded.

## 6. Conclusion

Each proximity space determines in a natural way a topological space with beneficial properties. Also, this theory possesses deep results, rich machinery, and tools. With the development of topology, the theory of proximity makes a great progress. Hence, the concept of proximity has been studied by many authors in both the fuzzy setting and the soft setting. In the present work, we mainly establish some properties of fuzzy soft proximity spaces in Katsaras's sense. We have shown that each fuzzy soft proximity determines a fuzzy soft topology by using fuzzy soft closure operator. Also, we present an alternative description of the concept of fuzzy soft proximity, which is called fuzzy soft *δ*-neighborhood. We believe that these results will help the researchers to advance and promote the further study on fuzzy soft topology to carry out a general framework for their applications in practical life.
